# Association of Fish and Omega-3 Fatty Acid Intake with Carotid Intima-Media Thickness in Middle-Aged to Elderly Japanese Men and Women: The Toon Health Study

**DOI:** 10.3390/nu14173644

**Published:** 2022-09-03

**Authors:** Koutatsu Maruyama, Salsabila Khairunnisa, Isao Saito, Takeshi Tanigawa, Kiyohide Tomooka, Satomi Minato-Inokawa, Madoka Sano, Misaki Takakado, Ryoichi Kawamura, Yasunori Takata, Haruhiko Osawa

**Affiliations:** 1 Laboratory of Community Health and Nutrition, Department of Bioscience, Graduate School of Agriculture, Ehime University, Matsuyama 790-8566, Japan; 2 Department of Public Health and Epidemiology, Faculty of Medicine, Oita University, Yufu 879-5503, Japan; 3 Department of Public Health, Juntendo University Graduate School of Medicine, Tokyo 113-8421, Japan; 4 Department of Diabetes and Molecular Genetics, Ehime University Graduate School of Medicine, Toon 791-0295, Japan

**Keywords:** fish, omega-3 fatty acid, intima-media thickness, Japanese

## Abstract

Fish and omega-3 fatty acid consumption is known to be beneficial for cardiometabolic health. However, the related evidence for individuals with a relatively higher intake of fish or omega-3 unsaturated fatty acids, e.g., Japanese individuals, is scarce. Therefore, this study aimed to examine the association of fish and omega-3 fatty acid intakes with the carotid intima-media thickness (C-IMT) in the Japanese population. In total, 1803 Japanese men and women aged 30–84 years without a history of myocardial infarction or angina pectoris were included in the study. The fish and omega-3 fatty acid intakes were estimated using food frequency questionnaires. The C-IMT was measured using ultrasound imaging, and the participants were classified into three groups: normal, moderate (1.1 to 1.4 mm of maximum C-IMT), and severely increased C-IMT (≥1.5 mm). Multinomial logistic regression models were used to calculate the odds ratio (OR) and 95% confidence intervals (95% CI) of the presence of moderately and severely increased C-IMT. The omega-3 fatty acid intake was shown to be associated with lower odds of severely increased C-IMT. The multivariable-adjusted OR (95%CI) was 0.55 (0.31–0.97; *p* for trend = 0.04). We also found a borderline significant negative association between fish intake and the presence of severely increased C-IMT. In conclusion, omega-3 fatty acid intake might protect against the development of atherosclerosis in the Japanese population.

## 1. Introduction

Fish is generally considered a healthy food item. A recent meta-analysis of 38 articles examined the association between fish intake and the risk of coronary heart disease with a prospective cohort design, and suggested that a higher fish intake was associated with a lower risk of coronary heart disease [1]. 

The primary pathophysiology behind the development of coronary heart disease is atherosclerosis [2] due to inflammatory processes involving modified lipoproteins, macrophages, T cells, and the cellular components of the vessel walls [3,4,5,6,7]. Research has shown that some of the nutrients capable of disrupting this process are omega-3 fatty acids, which are notably sourced from fish. Marine omega-3 fatty acids possess several benefits, such as the maintenance of the cell membrane fluidity, cardiometabolic health, and disruption of inflammatory processes, all of which could contribute to preventing atherosclerosis [8,9]. 

Carotid intima-media thickness (C-IMT) is a noninvasive surrogate marker of atherosclerosis [10]. An increased thickness may reasonably predict the incidence of cardiovascular disease [11]. Several observational studies have shown that a higher fish or omega-3 fatty acid intake is associated with a lower carotid intima-media thickness [12,13,14,15]. However, those studies were conducted in Western populations with relatively lower average consumption of fish and seafood [16] and higher mortality from coronary heart disease [17,18]. Although the evidence of this association in individuals with a relatively higher intake of fish or omega-3 fatty acids, e.g., Japanese and other Asian populations, is scarce, a recent meta-analysis showed a possible dose-response relationship between fish intake and a lower risk of coronary heart disease [1]. 

This study aimed to examine the association between fish and omega-3 fatty acid intake with C-IMT in the middle-aged and elderly Japanese population. Our a priori hypothesis was that a higher intake of fish and omega-3 fatty acids is associated with a lower C-IMT in the general Japanese population.

## 2. Materials and Methods

### 2.1. Participants

The Toon Health Study was a prospective cohort study carried out in Toon City, Ehime Prefecture, Japan since 2009. Data from 1843 men and women aged 30–84 years who participated from 2014 to 2018 were collected. This study used a cross-sectional design based on five-year follow-up survey data. The study details have been described elsewhere [19,20]. 

Participants with missing information from the carotid ultrasonography (n = 3) and food frequency questionnaire (n = 1) were excluded. Additionally, 36 individuals treated for myocardial infarction or angina pectoris were also excluded. A total of 606 men and 1197 women were included in this analysis. The Institutional Review Board of Ehime University Hospital approved the study protocol (#170511), and informed consent was obtained from each participant.

### 2.2. Measurement of Carotid Atherosclerosis

The right and left C-IMT values of the far wall were measured at the common carotid artery 10 mm proximal to the carotid bifurcation. Trained physicians conducted the ultra-sonographic scanning and interpreted the results using ultrasound imaging equipment (GM-72P00A; Panasonic Healthcare, Japan). The UK Biobank Study has shown the excellent validity and repeatability of C-IMT measurements obtained with this equipment [21]. In the present study, we calculated the mean of left and right maximum C-IMT values and categorized the participants into three groups based on the guidelines from the Japan Society of Ultrasonics in Medicine [22]: normal (<1.1 mm of maximum C-IMT), moderately increased (1.1 to 1.4 mm), and severely increased C-IMT (≥1.5 mm).

### 2.3. Dietary Assessment 

Food and nutrient intakes were assessed using a food frequency questionnaire (FFQ), for which the reasonable validity has been reported [23]. We asked questions regarding the consumption frequency of fish and fish products for each meal. The energy and nutrient intakes were estimated using the Standard Tables of Food Composition in Japan (Sixth revised edition) [24]. The omega-3 fatty acid intake is the sum of the alpha-linolenic, stearidonic, eicosatetraenoic, eicosapentaenoic, docosapentaenoic, and docosahexaenoic acid intakes. We also measured the validity of this FFQ in a subsample of participants from this study (14 men and 21 women). The Spearman rank correlation coefficients between the FFQ and the seven-day dietary records of fish and omega-3 fatty acids were 0.44 and 0.22, respectively. The density method was used to adjust all nutrient and food group intakes for energy intake. 

### 2.4. Assessment of Confounding Factors

The height in stocking-covered feet and weight in light clothing were measured. The BMI was calculated as the weight (kg)/height (m)^2^. The physicians asked for the participants’ medical history and current status of chronic diseases, including angina, myocardial infarction, diabetes mellitus, hypertension, and dyslipidemia. Blood pressure was measured twice after sitting for 5 min, and the mean of these measurements was used. Hypertension was defined as systolic blood pressure ≥140 mmHg or diastolic blood pressure of ≥90 mmHg, or current treatment with antihypertensive agents. The participants underwent a 75 g oral glucose tolerance test (OGTT), and the post-prandial serum glucose levels at baseline and 2 h after ingestion of 75 g of glucose were measured. Diabetes mellitus was defined as fasting serum glucose ≥7.0 mmol/L (126 mg/dL) or as ≥ 11.1 mmol/L (200 mg/dL) at 2 h post-prandial, or current treatment with hypoglycemic agents. Dyslipidemia was defined as fasting serum triglyceride ≥1.69 mmol/L (150 mg/dL), LDL-cholesterol ≥3.62 mmol/L (140 mg/dL), HDL-cholesterol ≤1.03 mmol/L (40 mg/dL), or current treatment with hypolipidemic agents.

In a self-administered questionnaire, the participants were asked about alcohol (current/nondrinker) and smoking habits (current/nonsmoker). Their physical activity was assessed using the Metabolic Equivalents of Task (MET) metric via the Japan Arteriosclerosis Longitudinal Study Physical Activity Questionnaire (JALSPAQ) [25]. A validation study for the JALSPAQ was conducted previously among 226 Japanese men and women ranging 20–83 years of age. In that study, the total energy expenditure (TEE) measured using the JALSPAQ was compared with the TEE measured using the doubly labeled water (DLW) method as a gold standard. The resting metabolic rate (RMR) was measured using the Douglas bag method. The TEE assessed by the JALSPAQ was moderately correlated with the DLW method (Spearman correlation = 0.742, *p* < 0.001; intraclass correlation coefficient = 0.648, *p* < 0.001). The JALSPAQ slightly underestimated the TEE compared with the DLW method by a difference of –1.15 ± 1.92 MJ/day [25].

### 2.5. Statistical Analysis

The participants were divided into sex-specific tertiles according to omega-3 fatty acid and fish intakes. Age- and sex-adjusted means and proportions of characteristics according to tertiles of omega-3 fatty acid and fish intakes were calculated using an analysis of covariance. The presence of a linear trend was tested using a multivariable regression analysis with the median value of each tertile. The multivariable-adjusted geometric means of the maximum C-IMT after the natural logarithmic transformation according to tertiles of omega-3 and fish intakes were calculated using an analysis of covariance, and the presence of a linear trend was tested using a multivariable regression analysis after adjustment for the following confounding factors: age, sex, BMI (continuous), physical activity (METs h/day; continuous), current drinking and smoking status (yes/no), energy (kcal/day; continuous) and energy-adjusted total vegetable and saturated fatty acid intakes (continuous), hypertension, diabetes mellitus, and dyslipidemia (yes/no). Finally, the odds ratio (OR) and 95% confidence intervals (95% CI) of the presence of moderately and severely increased C-IMT for second and third tertiles were compared with the respective lowest tertile after adjustment for the above confounding factors using a multinomial logistic regression model. Linear trends were tested using multinomial logistic regression models with the median value of each tertile. The probability values for statistical tests were two-tailed, and *p* < 0.05 was regarded as statistically significant. The SAS statistical package version 9.4 (Statistical Analysis Systems, Cary, NC, USA) was used for analyses.

## 3. Results

The fish and omega-3 fatty acid intakes were associated with a higher mean age and higher intakes of energy, total protein, and vegetables. Fish intake was positively associated with omega-3 fatty acid intake, and vice versa (Table 1). 

Table 2 shows age-, sex-, and multivariable-adjusted mean values of maximum C-IMT according to fish and omega-3 fatty acid intake tertiles, respectively. The fish and omega-3 fatty acid intakes were negatively associated with age- and sex-adjusted mean values of the maximum C-IMT. The association between the fish intake and maximum C-IMT remained after the adjustment for potential confounders. However, the association between the omega-3 fatty acid intake and maximum C-IMT did not reach significance. The multivariable-adjusted mean values of the maximum C-IMT for the lowest and highest tertiles of fish intake were 0.88 mm and 0.85 mm (*p* for trend = 0.048), respectively. The values for the lowest and highest tertiles of omega-3 fatty acid intake were 0.88 and 0.85 mm (*p* for trend = 0.054), respectively. These associations were not altered after further adjustment for dyslipidemia.

We calculated the ORs (95% CIs) for moderately and severely increased C-IMT according to tertiles of fish and omega-3 fatty acid intakes with a multinomial logistic regression model (Table 3). Although fish and omega-3 fatty acid intakes were not associated with moderate C-IMT, there was a significant negative association between omega-3 fatty acid intake and severely increased C-IMT. The multivariable OR (95% CIs) of severely increased C-IMT for the highest tertile compared with the lowest tertile of omega-3 fatty acid intake was 0.55 (0.31–0.97; *p* for trend = 0.04). We also found a borderline significant association between fish intake and severely increased C-IMT; the multivariable OR (95% CIs) of severely increased C-IMT for the highest tertile compared with the lowest tertile of fish intake was 0.59 (0.33–1.05; *p* for trend = 0.08). These associations were not altered after further adjustment for dyslipidemia. 

## 4. Discussion

In this study, we observed a significant negative association between omega-3 fatty acid intake and severely increased C-IMT, and a borderline significant negative association between fish intake and severely increased C-IMT. We also found that higher fish and omega-3 fatty acid intakes were associated with a lower maximum C-IMT.

Our findings are in line with the results from previous investigations. A community-based cross-sectional study among middle-aged Chinese individuals showed that higher levels of omega-3 fatty acids in the erythrocyte membrane were associated with lower odds of carotid artery wall thickening (>1.0 mm) [26]. Another study by Sekikawa et al. (2008) showed that differences in C-IMT between the Japanese population of Japan, and the Caucasian population of Allegheny County, Pennsylvania, were attenuated following an adjustment for marine omega-3 fatty acids [27]. 

Although we found an association between the omega-3 fatty acid intake and severely increased C-IMT only, there is a limited number of studies that have previously examined the associations of fish or an omega-3 fat intake with moderately and severely increased C-IMT separately. A potential reason for our detection of the association with severely increased C-IMT only may be that participants in this study included elderly individuals who had been assessed as at risk of increased C-IMT due to hypertension, dyslipidemia, and diabetes. Because the mean of the maximum C-IMT was relatively higher than in previous studies, we could not detect the association between the normal and moderate intake groups due to the low variability of the fish or omega-3 fat intake levels. On the other hand, a prospective study for patients with hypertension with higher mean levels of IMT showed that changes in the PUFA/SFA ratio in the RBC membranes induced by dietary intervention were inversely associated with changes in the IMT [13]. A higher omega-3 fatty acid intake may prevent the progression of IMT for individuals with atherosclerosis risk factors. Thus, we observed significant associations with severely increased C-IMT only. 

Omega-3 fatty acids reduce the atherosclerotic burden via two mechanisms: the improvement of the plasma lipid profile and prevention of the inflammation milieu in the atherosclerotic process. Omega-3 fatty acids have been approved as therapeutic agents for hypertriglyceridemia [28,29]. A meta-analysis of clinical trials assessing the diet’s impact on atherosclerosis risk in individuals with familial hypercholesterolemia also showed that the intake of omega-3 fatty acids was negatively associated with the triglyceride concentration [30]. The proposed mechanism behind the triglyceride-lowering effect observed after supplementation with omega-3 fatty acids was a reduction in VLDL-triglycerides synthesis [31] due to reduced fatty acid incorporation [32]. An increase in TG clearance has also been observed following omega-3 supplementation through increased lipoprotein lipase (LpL) activity [33] and endothelial binding [34]. In addition to its beneficial lipid profile improvements, previous studies have also shown that omega-3 fatty acids possess anti-inflammatory properties, such as inhibiting arachidonic acid metabolism [35], producing anti-inflammatory lipid mediators [36], and reducing tumor necrosis factor-kappa B (NFkB), resulting in lower expression levels of pro-inflammatory cytokines [37,38,39] and adhesion molecules [40,41,42]. These inflammatory processes are present in the early stages of atherosclerotic plaque development [43,44]; therefore, omega-3 fatty acids might be directly involved in preventing the development of atherosclerotic plaques through these pathways. 

This study had several strengths. First, the number of participants was large enough to detect the associations between fish and omega-3 fatty acid intakes with C-IMT. Second, the major potential confounding factors were measured and adjusted for by multivariable statistical models. However, the study was also subject to several limitations. First, the cross-sectional design of this study prevented insights into the causality. However, it should be noted that prospective cohort studies have shown that a higher omega-3 fatty acid intake is associated with a lower risk of increased C-IMT. Additionally, a meta-analysis of RCTs also showed that high-dose omega-3 fatty acid supplementation was associated with slower atherosclerotic plaque progression [45]. Therefore, a higher omega-3 fatty acid intake could be a prevention factor for developing increased C-IMT. Second, although our findings were similar and comparable to previous studies [26], the demographic characteristics (e.g., age, energy, and saturated fatty acid and vegetable intakes) per fish and omega-3 fatty acid intake tertile were also similar to a previous large-scale Japanese cohort study [17], whereby low-to-moderate validity of the fish and omega-3 fatty acid intakes as assessed by FFQ was observed in our subgroup population. Additionally, the estimated omega-3 fatty acid intake did not include intakes from dietary supplements. Thus, our findings might be restrictive, and a matter of concern might exist relating to the misclassification incurred in this FFQ.

Although the mean intake of fish and seafood [16] is relatively higher for the Japanese population than other populations, it has decreased gradually [16,46]. On the other hand, the mean intake of meat or meat products, mean levels of serum cholesterol levels, and incidence of coronary heart disease [47] for the Japanese population have increased over the past few decades. Thus, promoting the consumption of food items rich in omega-3 fatty acids, mainly fish, may effectively prevent atherosclerotic CVD, even for the Japanese population, who have relatively higher intakes of fish or omega-3 fatty acids. 

## 5. Conclusions

In this study, we observed a significant negative association between omega-3 fatty acid intake and severely increased C-IMT, and a borderline significant negative association between fish intake and severely increased C-IMT. We also found that higher fish and omega-3 polyunsaturated fatty acid intakes were associated with a lower maximum C-IMT. Our findings suggest that a higher omega-3 fat intake may prevent the development of atherosclerosis, even in populations with relatively high fish intake.

## Figures and Tables

**Table 1:** Demographic characteristics reported by fish and omega-3 fatty acid intake tertiles.

	Fish Intake				Omega-3 Fatty Acid Intake			
	T1 (Low)	T2	T3 (High)	*p* for Trend	T1 (Low)	T2	T3 (High)	*p* for Trend
N	601	601	601		601	601	601	
Age, year	55.7	63.4	67.1	<0.01	58.2	62.7	65.2	<0.01
Men, %	202 (33.6%)	202 (33.6%)	202 (33.6%)	-	202 (33.6%)	202 (33.6%)	202 (33.6%)	-
BMI, kg/m^2^	23.4	23.2	23.3	0.73	23.3	23.3	23.3	0.96
Dyslipidemia, %	51.3	51.4	49.0	0.31	54.2	48.8	48.7	0.05
Hypertension, %	39.6	39.7	41.5	0.43	40.8	39.4	40.5	0.90
Diabetes mellitus, %	15.0	13.5	13.0	0.34	14.5	14.7	12.4	0.28
Current smoker, %	7.5	8.8	7.0	0.57	8.7	8.2	6.4	0.09
Current drinker, %	56.1	58.2	58.2	0.43	53.8	60.5	58.2	0.12
Physical activity, METs h/day	35.1	35.1	35.4	0.22	35.0	35.2	35.3	0.31
Energy intake, kcal	1870.9	1895.5	1943.6	<0.01	1836.7	1908.5	1964.9	<0.01
Total fat, %energy	29.9	29.5	29.9	0.74	27.9	29.7	31.8	<0.01
Total protein, %energy	12.8	14.1	16.0	<0.01	13.1	14.1	15.6	<0.01
Omega-3 fatty acid, g/day	2.0	2.4	2.9	<0.01	1.8	2.4	3.1	<0.01
Omega-3 fatty acid, %energy	0.93	1.12	1.36	<0.01	0.86	1.12	1.43	<0.01
Saturated fatty acid, %energy	9.45	9.08	9.08	<0.01	9.16	9.15	9.31	0.14
Fish intake, g/1000 kcal	17.9	34.6	59.1	<0.01	23.7	35.5	52.0	<0.01
Vegetable intake, g/1000 kcal	111.7	127.0	138.3	<0.01	110.3	126.3	140.3	<0.01
Adjusted for age and sex.								
Men: Fish T1: <26.7; T2: 26.7–43.2; and T3: 43.2+ g/1000 kcal, omega-3 fat T1: <1.00; T2: 1.00–1.22; and T3: 1.22+ %energy.								
Women: Fish T1: <27.6; T2: 27.6–42.7; and T3: 42.8+ g/1000 kcal, omega-3 fat T1: <1.03; T2: 1.03–1.25; and T3: 1.25+ %energy.								

**Table 2:** Multivariable-adjusted means of maximum IMT reported via fish and omega-3 fatty acid intake tertiles.

	Fish Intake				Omega-3 Fatty Acid Intake			
	T1 (Low)	T2	T3 (High)	*p* for Trend	T1 (Low)	T2	T3 (High)	*p* for Trend
N	601	601	601		601	601	601	
Age- and sex adjusted means, mm	0.82	0.81	0.79	0.04	0.82	0.80	0.79	0.04
Multivariable-adjusted means, mm *	0.88	0.87	0.85	0.048	0.88	0.86	0.85	0.054
Multivariable-adjusted means, mm †	0.88	0.87	0.85	0.049	0.88	0.86	0.85	0.06
All mean values are expressed as geometric means.								
* Adjusted for age, sex, physical activity (METs h/day), BMI, current drinking and smoking status, energy, vegetable and saturate fatty acid intakes, hypertension, and diabetes.								
† Further adjusted for dyslipidemia.								
Men: Fish T1: <26.7; T2: 26.7–43.2; and T3: 43.2+ g/1000 kcal, omega-3 fat T1: <1.00; T2: 1.00–1.22; and T3: 1.22+ %energy.								
Women: Fish T1: <27.6; T2: 27.6–42.7; and T3: 42.8+ g/1000 kcal, omega-3 fat T1: <1.03; T2: 1.03–1.25; and T3: 1.25+ %energy.								

**Table 3:** Odds ratios (OR) and 95% confidence intervals (95% CI) of moderately and severely increased C-IMT from a multinomial logistic regression model, reported via fish and omega-3 fatty acid intake tertiles.

	Fish Intake				Omega-3 Fatty Acid Intake			
	T1 (Low)	T2	T3 (High)	*p* for Trend	T1 (Low)	T2	T3 (High)	*p* for Trend
N	601	601	601		601	601	601	
Moderately increased C-IMT, n (%)	26 (4.3%)	41 (6.8%)	42 (7.0%)		30 (5.0%)	34 (5.7%)	45 (7.5%)	
Age- and sex adjusted OR	1.00	1.04 (0.61–1.76)	0.86 (0.50–1.46)	0.48	1.00	0.87 (0.52–1.46)	1.02 (0.62–1.67)	0.88
Severely increased C-IMT, n (%)	27 (4.5%)	32 (5.3%)	32 (5.3%)		31 (5.2%)	33 (5.4%)	27 (4.5%)	
Age- and sex adjusted OR	1.00	0.75 (0.43–1.31)	0.59 (0.34–1.03)	0.07	1.00	0.79 (0.47–1.33)	0.56 (0.33–0.98)	0.04
Multivariable-adjusted OR * for moderately increased C-IMT	1.00	1.05 (0.62–1.79)	0.87 (0.50–1.52)	0.54	1.00	0.90 (0.53–1.53)	1.10 (0.65–1.84)	0.67
Multivariable-adjusted OR * for severely increased C-IMT	1.00	0.73 (0.42–1.29)	0.59 (0.33–1.05)	0.08	1.00	0.78 (0.45–1.33)	0.55 (0.31–0.97)	0.04
Multivariable-adjusted OR † for moderately increased C-IMT	1.00	1.05 (0.62–1.80)	0.88 (0.51–1.52)	0.55	1.00	0.91 (0.53–1.54)	1.10 (0.66–1.86)	0.65
Multivariable-adjusted OR † for severely increased C-IMT	1.00	0.74 (0.42–1.29)	0.59 (0.33–1.05)	0.09	1.00	0.78 (0.46–1.34)	0.55 (0.31–0.98)	0.045
* Adjusted for age, sex, physical activity (METs h/day), BMI, current drinking and smoking status, energy, vegetable and saturated fatty acid intake, hypertension, and diabetes.								
† Further adjusted for dyslipidemia.								
Men: Fish T1: <26.7; T2: 26.7–43.2; and T3: 43.2+ g/1000 kcal, omega-3 fat T1: <1.00; T2: 1.00–1.22; and T3: 1.22+ %energy.								
Women: Fish T1: <27.6; T2: 27.6–42.7; and T3: 42.8+ g/1000 kcal, omega-3 fat T1: <1.03; T2: 1.03–1.25; and T3: 1.25+ %energy.								

## Data Availability

Not applicable.
